# Supplementary material for: Health-Related Quality of Life (HRQoL) in Neuroendocrine Tumors: A Systematic Review
Source: Cancers (Basel). 2022 Mar 10;14(6):1428. doi: 10.3390/cancers14061428 (PMC8946839; doi:10.3390/cancers14061428)
Supplement: Supplementary file 1 [file cancers-14-01428-s001.zip › cancers-1603860-supplementary.pdf]

Table S1. Prospective and Observational Studies in NET Patients with Reporting of HRQoL Outcome Measures.

Study	Type of Study	Sample Size	Treatment	Tumor Sub-type	HRQoL Measure
O'Toole et al., 2000[35]	Prospective study	33	octreotide followed by lanreotide	GI NETs	ISPN
Jacobsen & Hanssen, 1995[36]	Prospective Study	11	Octreotide vs. placebo	GI NETs	GHQ-30, PAIS
Larsson et al., 2001[37]	Prospective study	24	interferon; somatostatin analog; interferon + somatostatin analog	GI NETs	QLQ-C30
Kwekkeboom et al., 2003[38]	Prospective study	35	^{177}Lu-DOTATATE	GI NETs	QLQ-C30
Pasieka et al., 2004[39]	Prospective study	24	^{131}I- meta- iodbenzylguanidin; ^{111}In-octreotide	NETs	adhoc questionnaire
Teunissen et al., 2004[40]	Prospective study	50	^{177}Lu-DOTATATE	NETs	QLQ-C30
Fröjd et al., 2007; 2009[41,42]	Prospective study	36	interferon; octreotide; interferon + octreotide; chemotherapy; octreotide + chemotherapy; no treatment	NETs	QLQ-C30
Larsson & Janson, 2008[43]	Prospective study	18	Interferon + erythropoietin	GI NETs	QLQ-C30, FACT-An
Kalinowski et al., 2009[44]	Prospective study	9	^{90}Y-microspheres	NETs	QLQ-C30, QLQ-LMC21
Haugland et al., 2013[45]	Prospective study	37	Multidisciplinary educational intervention	GI NETs	SF-36
Spolverato et al., 2015[46]	Prospective study	85	surgical vs. nonsurgical treatment	NETs	self-constructed questionnaire
Larsson et al., 1998[47]	Cross-sectional study	17	interferon; somatostatin analog; interferon + somatostatin analog	NETs	QLQ-C30
Larsson, Sjöden et al., 1999[48]	Cross-sectional study	119	interferon + octreotide; interferon; octreotide; chemotherapy; radiation; omeprazole; no treatment	NETs	QLQ-C30
Larsson, von Essen et al., 1999[49]	Cross-sectional study	99	interferon; somatostatin analog; interferon + somatostatin analog	NETs	QLQ-C30
von Essen et al., 2002[50]	Cross-sectional study	85	interferon; somatostatin analog; interferon + somatostatin analog	GI NETs	QLQ-C30
Berglund et al., 2003[51]	Cross-sectional study	36	Not specified	GI NETs	HADS, IES, LOT, SF-36
Larsson et al., 2003[52]	Cross-sectional study	19	interferon; somatostatin analog; interferon + somatostatin analog	NETs	interviews
Davies et al., 2006[53]	Cross-sectional study	180	Not specified	NETs	QLQ-GI.NET21, QLQ-C30
Larsson et al., 2007[54]	Cross-sectional study	83	Interferon/octreotide; chemotherapy; radiation; omeprazole; no treatment	NETs	QLQ-C30
You et al., 2007[55]	Cross-sectional study	50	surgery	GI NETs	QLQ-C30, GQLI
Haugland et al., 2009[56]	Cross-sectional study	96	interferon, somatostatin analog; interferon + somatostatin analog; chemotherapy; no treatment	GI NETs	SF-36
Pezzilli et al., 2009[57]	Cross-sectional study	44	somatostatin analog; somatostatin analog + other; no treatment	GI NETs	SF-12, GHQ-12
van der Horst-Schrivers et al., 2009[58]	Cross-sectional study	43	somatostatin analog; interferon; somatostatin analog + interferon	GI NETs	QLQ-C30

Pezzilli et al., 2010[59]	Cross-sectional study	44	somatostatin analog; interferon; chemotherapy; somatostatin analog + other; no treatment	GI NETs	SF-12
Vinik et al., 2011[60]	Cross-sectional study	29	Not specified	NETs	Norfolk QOL-NET
Beaumont et al., 2012[61]	Cross-sectional study	663	surgery; surgery + SSA; other	NETs	SF-36, PROMIS global health short form, PROMIS-29
Petzel et al., 2012 [62]	Cross-sectional study	240	Not specified	GI NETs	FACT-Hep
Pavel et al., 2013[63]	Cross-sectional study	126	everolimus	GI NETs	QLQ-C30, QLQ-GI.NET21
Yadegarfar et al., 2013[64]	Cross-sectional study	253	Not specified	NETs	QLQ-GI.NET21, QLQ-C30
Gelhorn et al., 2016[65]	Cross-sectional study	96	telotristat	GI NETs	QLQ-C30, QLQ-GI.NET21
Haugland et al., 2016[66]	Cross-sectional study	196	not specified	GI NETs	SF-36
Pearman et al., 2016[67]	Cross-sectional study	11	surgery; surgery + SSA; other	NETs	SF-36, PROMIS global health short form, PROMIS-29
Ruszniewski et al., 2016[68]	Cross-sectional study	273	lanreotide autogel	NETs	QLQ-C30, QLQ-GI.NET21
Halperin et al., 2018[69]	Cross-sectional study	117	somatostatin analog; surgical procedure; chemotherapy; interferon; targeted therapy	NETs	FACT-G, PROMIS-29
Marinova et al., 2018[70]	Cross-sectional study	68	PRRT	GI NETs	QLQ-C30
Milanetto et al., 2018[71]	Cross-sectional study	100	surgery	GI NETs	QLQ-C30, QLQ-GI.NET21

Abbreviations: NET= Neuroendocrine tumors, HRQoL= Health Related Quality of Life, GI= Gastrointestinal, vs.= versus, IFN= interferon alpha; LAR = long-acting release, 5FU= 5-Flurouracil, SSA= Somatostatin Analogue, PRRT= peptide receptor radionuclide therapy, EORTC QLQ-30= European Organization for Research and Treatment of Cancer Quality of Life Core Questionnaire, EORTC QLQ GI.NET21= NET specific EORTC QoL questionnaire, FACT-G = Functional Assessment of Cancer Therapy-General, GHQ= General Health Questionnaire, PROMIS- Patient-Reported Outcome Measurement Information System, SF=Item Short Form Health Survey, HADS=Hospital Anxiety and Depression Scale, IES= Impact of Event Scale, LOT=Life Orientation Test, FACIT= The Functional Assessment of Chronic Illness Therapy.
